# Study on the Selection of the Targets of Esophageal Carcinoma and Interventions of Ginsenosides Based on Network Pharmacology and Bioinformatics

**DOI:** 10.1155/2020/4821056

**Published:** 2020-06-24

**Authors:** Xin Yang, Yahui Li, Haibing Qian

**Affiliations:** Guizhou University of Traditional Chinese Medicine, Guiyang 550025, China

## Abstract

**Background:**

Esophageal carcinoma (ESCA) is not only a threat to people's health but also the sixth most common cause of cancer-related mortality worldwide.

**Methods:**

In this study, the key targets of ESCA are screened through GeneCards and DisGeNET databases combined with the Gene Expression Omnibus (GEO) database (GSE1420 and GSE20347). Then, data associated with ESCA samples are downloaded from The Cancer Genome Atlas (TCGA) database for integrated analysis. Moreover, the effect of epithelial cell adhesion molecule (EpCAM) expression on the survival of patients with ESCA is evaluated by Kaplan–Meier and Cox analyses. The virtual screening is carried out using a Suflex-Dock molecular docking module. The chemical components, which have been well bound to EpCAM, are screened out based on a total score >5 as a threshold. Ginsenosides and EpCAM are analyzed by LigPlot + v.2.2 software to identify the binding sites.

**Results:**

Four ESCA targets are obtained from GeneCards, DisGeNET, and GEO databases. In this study, it is found that high EpCAM expression is associated with histologic grade, stage, patient age, *N* classification, *T* classification, and radiation therapy. The Kaplan–Meier curves for overall survival also show that the higher expression of EpCAM is associated with worse outcomes in patients with ESCA. Univariate and multivariate Cox analyses indicate that EpCAM mRNA expression might be a useful biomarker for ESCA(*P* < 0.05). Molecular docking technology suggests that ginsenoside Rg3 and ginsenoside Rh2 can easily establish good docking modes and have a high affinity with EpCAM. The 6′-hydroxyl and 6″-hydroxyl on the 3-glycosyl of ginsenoside Rg3 are prone to form hydrogen bonds (Lys151 and Lys221) with the active sites of EpCAM ligand binding domain. The hydroxyl groups on the 12 sites of the ginsenoside Rh2 glycoside framework are found to have hydrogen bonding with Leu240. The formation of hydrogen bonds plays an important role in binding of ginsenoside Rg3 and ginsenoside Rh2 to EpCAM, as well as the stability of EpCAM conformation.

**Conclusion:**

EpCAM may be determined as a potential biomarker for early diagnosis and prognosis of ESCA. Ginsenoside Rg3 and ginsenoside Rh2 have potential antiesophageal cancer activities. This experiment provides a reference for the study of the chemical compositions of ginsenosides in the treatment of esophageal cancer.

## 1. Introduction

Esophageal carcinoma (ESCA) is a type of malignancy prevalent worldwide consisting of esophageal adenocarcinoma (EAC) and esophageal squamous cell carcinoma (ESCC) [1]. ESCA is not only a threat to people's health, but it also is the sixth most common cause of cancer-related deaths worldwide [2]. Besides, ESCA has higher morbidity and mortality. There is a poor prognosis of ESCA; for example, the 5-year survival rate in a nonmetastatic environment is between 20% and 35%. Even if the operation goes well, the patient might die from the complete resection of the primary tumor and multimode treatment [3, 4]. ESCA is usually caused by a malignant tumor that originates in the esophageal epithelium. ESCA mainly manifests as squamous cell carcinoma and adenocarcinoma. However, some rare syndromes present as mucoepidermoid carcinoma, small cell carcinoma, neuroendocrine tumors, and adenosquamous carcinoma [5]. It is difficult to detect early ESCA by conventional endoscopy and radiological examination, which allows the malignant progression of ESCA via distant metastases through the lymphatic system. Furthermore, even though an ESCA is detected, the developments in endoscopic imaging, ablation, and resection techniques have resulted in the dependence of endoscopy and have limited its role in the therapeutic model [6]. Therefore, it is of great clinical significance to identify reliable biomarkers for the diagnosis and prognosis of ESCA. In this study, the key targets of ESCA are screened by network pharmacology combined with bioinformatics. Then, data associated with esophageal carcinoma samples are downloaded from TCGA database for integrated analysis.

Epithelial cell adhesion molecule (EpCAM) is a transmembrane glycoprotein originally described by Koprowski et al. [7]. It is considered a reliable surface binding site for pure cell adhesion molecules and therapeutic antibodies. EpCAM is a cancer stem cell (CSC) marker, which is expressed in various epithelial carcinomas comprising ESCA. Recent research has shown a clear correlation between human cancers and high expression of EpCAM of human cancers [8, 9]. High expression of EpCAM in primary tumors is often associated with more aggressive phenotypes. It also has a negative impact on the patient's prognosis [10]. Some carcinoma-associated antigen encoded by EpCAM is detected on healthy epithelial cells and gastrointestinal carcinomas [11, 12]. Therefore, it provides evidence that EpCAM is important for the diagnosis and treatment of these types of cancers.

**Figure 1:**
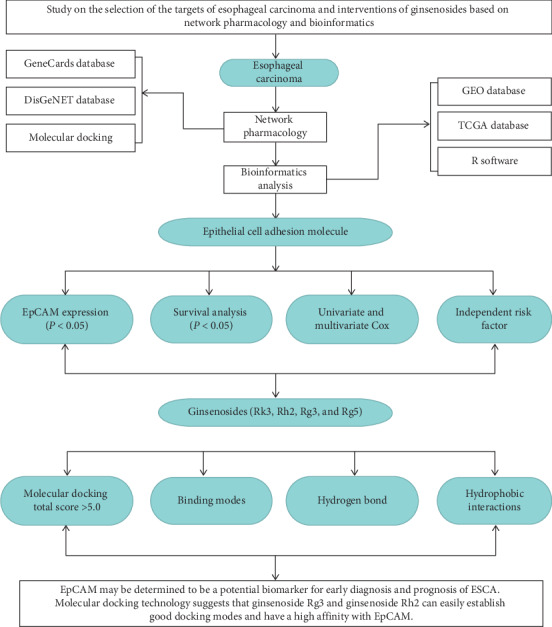
Flowchart for network pharmacology and bioinformatics analysis of publicly available data.

Ginsenosides are mainly extracted from the Araliaceae plants, which are called the prototype ginsenosides. Their transformation products are named rare ginsenosides. More and more studies have shown that the rare ginsenosides have a strong antitumor effect and a wide range of antitumor mechanisms [13]. They can achieve the therapeutic effect through many ways, such as direct actions on cancer cells, inhibition of the tumor growth by indirect induction of apoptosis, and enhancement of immunity [14]. These highlight the ginsenosides' functional advantages and they are expected to become an important drug in the treatment of cancer. At present, there are few reports about the effect of ginsenosides on esophageal cancer. Nonetheless, the existing studies have found that the rare ginsenoside Rk3, ginsenoside Rg5, ginsenoside Rh2, and ginsenoside Rg3 have a certain therapeutic effect in esophageal cancer [15–18]. Therefore, ginsenoside Rk3, ginsenoside Rg5, ginsenoside Rh2, and ginsenoside Rg3 have been selected as the research objects in the present study to investigate the interaction between them and EpCAM, a therapeutic target of esophageal cancer, at the molecular level. The virtual screening is carried out using a Surflex-Dock molecular docking module. The chemical components, which have been well bound to EpCAM, are screened out based on a total score >5 as a threshold. Ginsenosides and EpCAM are analyzed by LigPlot + v.2.2 software to identify the binding sites. The workflow for network pharmacology and bioinformatics analysis of publicly available datasets is shown in Figure 1.

## 2. Materials and Methods

### 2.1. Target Screening for ESCA

The targets of ESCA are obtained from GeneCards Human gene database (https://www.genecards.org/) and DisGeNET (http://www.disgenet.org/search). We searched “esophageal carcinoma” in GeneCards and DisGeNET. Common targets for GeneCards and DisGeNET were obtained by using Venn diagram package (FunRich 3.1).

### 2.2. GEO Data Download and Preprocessing

High-sequence data of GSE1420 (GPL96, Affymetrix Human Genome U133A Array) and GSE20347 (GPL571, Affymetrix Human Genome U133A 2.0 Array) are collected from the Gene Expression Omnibus (GEO) database (https://www.ncbi.nlm.nih.gov/geo/) by using the following keywords: “esophageal carcinoma,” “*Homo sapiens*,” and “gene expression data” [19]. GSE1420 comprises eight ESCA tissues and eight adjacent nontumor tissues. GSE20347 includes 17 ESCA tissues and 17 adjacent nontumor tissues. Briefly, the CEL format files are used as input. Background correction and normalization are conducted through using the robust multichip average function implemented in the affy package in R software (version 3.5.1).

### 2.3. Identification of Differentially Expressed Genes

In the present study, the LIMMA package was used for the identification of differentially expressed genes (DEGs) between the ESCA and normal tissues. The Benjamini-Hochberg procedure was introduced to reduce the false discovery rate (FDR) in multiple comparisons. The DEGs' cutoff value was set as |log 2 Fold Change>2| and FDR as <0.05 [20]. The common DEGs for GSE1420 and GSE20347 were obtained by using the Venn diagram package (FunRich 3.1).

### 2.4. TCGA Data Download and Preprocessing

Genome-wide transcriptome profiles of 160 ESCA tissues and 11 adjacent nontumor tissues are downloaded from The Cancer Genome Atlas (TCGA) database (https://cancergenome.nih.gov/) by using the following keywords: “esophageal carcinoma,” “*Homo sapiens*,” “gene expression quantification,” and “HTSeq-FPKM.” The clinical data of 183 ESCA patients are downloaded from TCGA database by searching the following keywords: “esophageal carcinoma,” “clinical,” and “BCR XML.” Clinical data include patients' age, alcohol consumption history, gender, survival status, TNM classification, and pharmaceutical as well as histologic grade. All the data are processed by R software (version 3.5.1).

### 2.5. Statistical Analysis

The methods outlined by Jiao et al. is followed by the researchers [21]. The expression of EpCAM, MMP1, SPP1, and CRNN in patients in the TCGA-ESCA dataset is evaluated by using plot points. The overall survival (OS) is compared between the high EpCAM, MMP1, CRNN, and SPP1 expression groups through the method of Kaplan–Meier analysis. This analysis is performed based on the expression and clinical data of the TCGA database. Perl is used to merge the data. EpCAM, MMP1, SPP1, and CRNN are divided into two strata based on the expression level and median value. The chi-square and Fisher's exact tests are applied to identify the correlation between EpCAM mRNA expression and the clinical features of ESCA. A univariate Cox analysis is performed to select potential prognostic factors. A multivariate Cox analysis is conducted to verify the correlation between EpCAM expression and survival along with other clinical features. *P* < 0.05 is considered statistically significant.

### 2.6. Compounds and EpCAM Molecular Docking and Interaction

Molecular docking is a powerful computation tool that can be used to predict the interaction energy between a receptor and a ligand. In order to do so, it determines the orientation of the ligand that would form the lowest energy complex within the receptor's binding pocket. In this docking assay, the 2D structures of ginsenoside Rk3, ginsenoside Rh2, ginsenoside Rg3, and ginsenoside Rg5 are downloaded from the PubChem database. Human epithelial cell adhesion molecule (hEpCAM, PDB ID:4MZV:1.865 Å) [22] receptors are retrieved from the Protein Data Bank (PDB). The SYBYL 2.1.1 program is used to evaluate the binding potential targets of EpCAM and ginsenosides. The Surflex-Dock scores (total score) are expressed in −log10 (Kd) units [23]. The ginsenoside and EpCAM interactions are observed and analyzed through using the LigPlot + v.2.2 software [24].

## 3. Results

### 3.1. Screening of Intersections of Genes in ESCA

**Figure 2:**
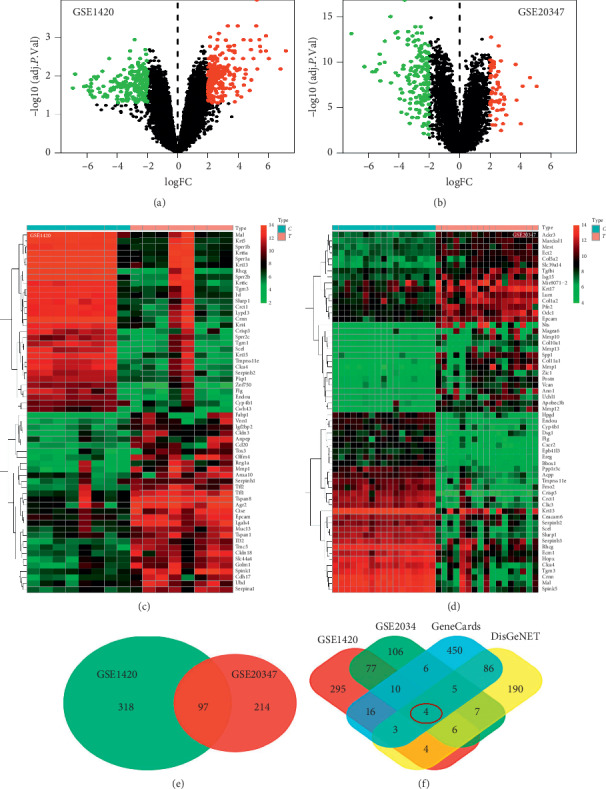
DEGs expression profile in the GEO, GeneCards, and DisGeNET databases. (a) Volcano plots showed the number of DEGs identified from GSE1420. (b) Volcano plots showed the number of DEGS identified from GSE20347. (c) Heat map of DEGs in GSE1420 (top 60). Hot spots ranged from red (high expression) to green (low expression). (d) Heat map of DEGs in GSE20347 (top 60). Hot spots ranged from red (high expression) to green (low expression). (e) Venn diagram demonstrates the intersections of genes between GSE1420 and GSE20347. (f) Venn diagram demonstrates the intersections of genes between GEO, GeneCards, and DisGeNET databases.

Two GEO datasets are downloaded, preprocessed, and merged into a global dataset that contains 25 esophageal cancer and 25 normal samples (Table 1). The functions of GSE1420 and GSE20347 are normalized so as to make intragroup comparisons of the measurements of each time/group under various experimental conditions. The distributions of DEGs are presented in volcano plots and heat map (Figures 2(a)–2(d)). A total of 415 DEGs of GSE1420 and 221 DEGs of GSE20347 are identified by the LIMMA package. 97 DEGs are identified in the two samples in considering the intersections (Figure 2(e)). The ESCA targets are obtained from the GeneCards and DisGeNET databases, and then 580 (relevance score >10%) and 305 (*P* < 0.05) DEG targets are retrieved, respectively. Later, four ESCA disease targets (EpCAM, MMP1, SPP1, and CRNN) are obtained by combining GSE1420 and GSE20347 (Figure 2(f)).

### 3.2. Characteristics of the Study Population

The clinical data of 183 ESCA patients are downloaded from TCGA database comprising patients' age, alcohol consumption history, gender, survival status as well as TNM classification, pharmaceutical, histologic grade, clinical stage, and radiation therapy of ESCA (Table 2).

### 3.3. Screening Key Targets

**Figure 3:**
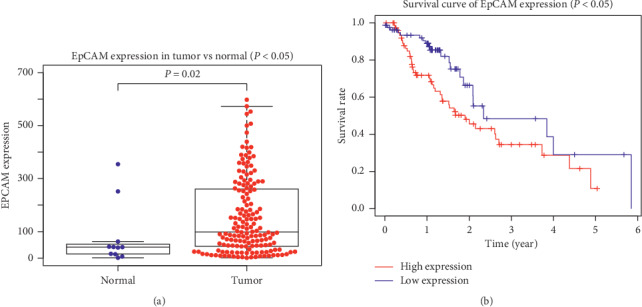
EpCAM expression and survival analysis in ESCA. (a) EpCAM expression in ESCA (blue dot: low expression; red dot: high expression). (b) The blue line represents ESCA patients with relatively low EpCAM mRNA expression, and the red line represents ESCA patients with high expression of the aforementioned gene.

There is a comparison between EpCAM expression in ESCA and normal tissues. The results of comparison indicates that EpCAM expression is elevated in ESCA (*P* < 0.05). Whether the identified prognostic markers have a value for predicting patient survival is evaluated; for example, EpCAM, overexpressed in ESCA, shows a negative correlation with patient survival (Figure 3). In other words, patients with a higher expression of EpCAM has worse overall survival (*P* < 0.05). The expression of CRNN is normal, and ESCA has no statistical significance as well (*P* > 0.05), according to Figure S1. MMP1 and SPP1 expression is elevated in ESCA. The expression levels of MMP1, SPP1, and CRNN show that there is no association between them and the prognosis of ESCA patients for overall survival (*P* > 0.05). In the same way, we conducted no further analysis on those genes, but concentrated on the analysis of EpCAM.

### 3.4. High EpCAM Expression in ESCA

**Figure 4:**
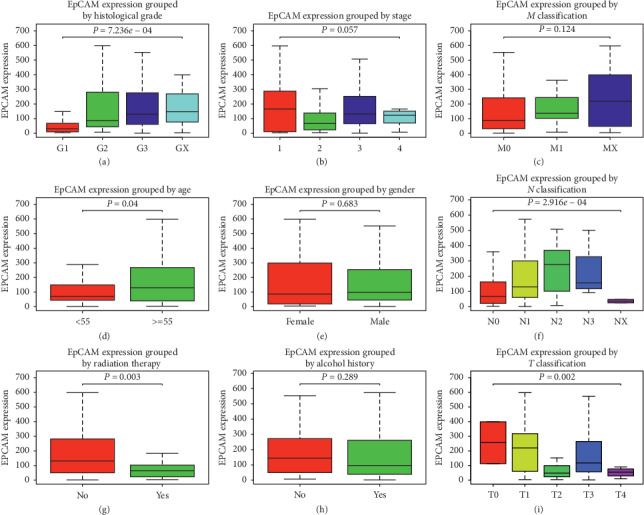
EpCAM expression in ESCA. EpCAM expression was compared between normal tissues and ESCA tissues as well as according to the histologic grade, cancer stage, *M* classification, patient age, gender, *N* classification, treatment with radiation, alcohol consumption history, and *T* classification.

EpCAM expression in ESCA and normal tissues is compared (Figure 4).The differences in EpCAM expression are observed according to histologic grade (*P*=7.236*e* − 4), stage (*P*=0.018), patient age (*P*=0.04), *N* classification (*P*=2.916*e* − 4), radiation therapy (*P*=0.003), and *T* classification (*P*=0.002).

### 3.5. High EpCAM Expression Is an Independent Risk Factor for Overall Survival among ESCA Patients

Univariate and multivariate Cox (Figure S2) analyses show that EpCAM expression is an independent risk factor for overall survival (OS) among ESCA patients (hazard ratio [HR] = 1.00, 95% confidence interval (CI): 1.00–1.01, *P*=0.01; Table 3).

### 3.6. Molecular Docking and Interaction

**Figure 5:**
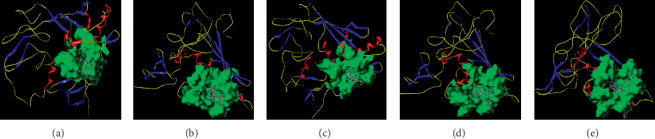
Docking modes of ginsenosides with EpCAM. (a) Original ligand (DMU301), (b) ginsenoside Rg3, (c) ginsenoside Rh2, (d) ginsenoside Rg5, and (e) ginsenoside Rk3.

EpCAM is inputted into the SYBYL 2.1.1 program for molecular docking verification. Ginsenoside Rk3, ginsenoside Rh2, ginsenoside Rg3, and ginsenoside Rg5 are also inputted into the docking program (Table 4). The crystal structure of EpCAM is found to contain an original ligand, with active pockets determined by the original crystal structure A/DMU301. The docking results provide a similarity value that ranges between 0 and 1, whereby the larger the value is, the more similar the molecular conformation is. The similarity value for the docking and original conformations is 0.75, which indicates a good overlap and good accuracy of docking process. The total score parameter is used as an evaluation index of the molecular docking results. A total score ≥5.0 indicates that active molecules have strong binding activity with EpCAM. A total score ≥7.0 suggests that active molecules have an even stronger binding activity with EpCAM. CH_4_, C=O, and N-H are used as molecular probes to determine the cavity of the receptor and perform the docking. The results of the docking indicate that ginsenoside Rg3 and ginsenoside Rh2 had a strong binding activity with EpCAM. In addition, the glycosyl chains of the ginsenoside Rg3, ginsenoside Rk3, and ginsenoside Rg5 are found to bind to residues outside the active pocket by hydrogen bonding. The hydroxyl groups on the ginsenoside Rh2 glycoside skeleton are found to combine with the residues in the active pocket by hydrogen bonding, the total spatial structure of which is reasonable. However, the level of exposure in the active pocket is not exactly the same, and they interact with the molecules in the pocket (Figure 5). The analysis of interactions between ginsenoside Rk3, ginsenoside Rh2, ginsenoside Rg3, and ginsenoside Rg5 and EpCAM are performed by the LigPlot + v.2.2 software (Figure S3). In ginsenoside Rg3, 6′-hydroxyl on three glycosyl groups forms hydrogen bonding forces with Lys151, and 6″-hydroxyl on three glycosyl groups forms hydrogen bonding forces with Lys221 (dashed line, Figure S3(a)). The hydroxyl groups on the 12 sites of ginsenoside Rh2 glycoside framework are found to have a hydrogen bonding with Leu240 (dashed line, Figure S3(b)). In ginsenoside Rg5, 6′-hydroxyl on three glycosyl groups forms hydrogen bonding forces with Lys151, and 6″-hydroxyl on three glycosyl groups forms hydrogen bonding forces with Lys241 (dashed line, Figure S3(c)). In ginsenoside Rk3, the glycosyl groups on six sites forms hydrogen bonding forces with both Lys151 and Asp241 (dashed line, Figure S3(d)).

## 4. Discussion

With the extensive application of gene chip technologies, abundant expression profile information, and screening of DEGs, biomarkers in tumor tissues could be detected efficiently by integrating publicly available datasets. Multidisciplinary cross-integration contributes to the rapid development of bioinformatics [25]. The GEO database collects large amounts of genomics data such as gene chips, filters, and serial analyses of gene expressions, which are submitted by researchers from all over the world [19]. This GEO database facilitates genetic studies and provides important online resources for the reintegration and in-depth exploration of gene expression data [26]. TCGA is a database of cancer gene information that includes gene expression data, cancer mutation profiles, and related clinical information [27]. The above-mentioned databases represent valuable resources for studying the appearance, development, and prognostic status of cancer. In this study, we also integrate molecular docking technology, which can predict the binding degree of small molecules to targets and facilitate the screening of active components in Chinese medicines. The screening of prognostic genes for ESCA has been addressed in previous studies [28]. On the contrary, this study combines network pharmacology with bioinformatics to screen four ESCA targets. Moreover, it is found that the EpCAM gene may be a potential target for the early diagnosis and prognosis of ESCA by deep mining TCGA clinical data.

By reviewing the study characteristics, the EpCAM gene contains 314 amino acids with a relative molecular weight of 40 kDa. Given today's resources, the characteristics of EpCAM that act as CSC markers can be considered [29, 30]. It has been proved that CSC subpopulations can initiate cancer development, promote cancer metastasis and drug resistance, and even lead to recurrence of ESCA [31]. Furthermore, in ESCA patients, it is known that the expression of the EpCAM is dynamic. In the formation of tumors, high expression is associated with the proliferation of cells, whereas low expression is related to migratory and invasive phenotypes of ESCA cells [32]. These two points show that the expression of EpCAM provides the conditions necessary for the growth of ESCA. In the present study, a high EpCAM expression in ESCA is observed, which is consistent with other findings related to the EpCAM expression in tumors. It is found that distinct histologic grades and a survival status are associated with EpCAM expression, which suggests a possible relationship between EpCAM expression and survival in ESCA. Collectively, our data demonstrate genome-wide transcriptional regulation by EpCAM and suggest target genes as biomarker candidates for EpCAM-associated ESCA.

Ginseng saponins are characterized by many positive biological activities, including antitumor, antiradiation, and antiaging activities [33, 34]. Modern studies have shown that ginsenosides have antitumor effects and relatively minor side effects. Ginsenoside Rh2 [15], ginsenoside Rg3 [16], ginsenoside Rk3 [17], and ginsenoside Rg5 [18] have been proven to play important roles in ESCA. Many studies have confirmed that the anticancer effect of ginsenosides is mainly related to cell apoptosis, cell autophagy, or cycle arrest. Ginsenoside Rh2 and ginsenoside Rg3, in particular, exhibit the strongest ability to inhibit cancer cells and have low toxicity to normal cells [35, 36]. In this study, ginsenoside Rg3, ginsenoside Rh2, ginsenoside Rg5, and ginsenoside Rh2 are found to form stable hydrogen bonds with Lys151, Lys221, Leu240, and Asp241 amino acid residues to increase the binding stability and then are concurrently subjected to hydrogen bond forces and strong hydrophobic interaction at the binding sites with Leu233, Asp232, Leu242, Pro244, Val220, and Asp219 hydrophobic residues. These two active molecules, ginsenoside Rg3 and ginsenoside Rh2, have strong binding activity with EpCAM targets, with docking scores higher than 5.0. In ginsenoside Rg3, 6′-hydroxyl on three glycosyl groups formed hydrogen bonding forces with Lys151, and 6″-hydroxyl on three glycosyl groups formed hydrogen bonding forces with Lys221. The hydroxyl groups on the 12 sites of the ginsenoside Rh2 glycoside framework were found to have hydrogen bonding with Leu240. The formation of hydrogen bonds plays an important role in binding of ginsenoside Rg3 and ginsenoside Rh2 to EpCAM, as well as the stability of EpCAM conformation.

This study, by the integration of content from multiple disciplines, confirmed that the high expression level of EpCAM is associated with poor overall survival of patients with ESCA. This finding is of great significance to the clinical diagnosis and treatment of ESCA and also provides a theoretical basis for clarification of the role of EpCAM in ESCA. This study provides novel insights into the molecular mechanism of ESCA and may serve as a reference for clinical studies in ESCA.

## 5. Conclusion

This study screens four ESCA targets combining network pharmacology with bioinformatics. Then, it is found that EpCAM may be a potential biomarker for early diagnosis and prognosis of ESCA through the deep mining of TCGA clinical data. Molecular docking technology indicates that ginsenoside Rg3 and ginsenoside Rh2 can easily establish good docking modes and have a high affinity with EpCAM. Moreover, it will further provide references for virtual screening of ginsenosides. There are some limitations in this study. Relevant clinical and basic experiments should be conducted in the future to verify the stability and practicability of the target.

## Figures and Tables

**Table 1:** The detail information of four GEO datasets.

GEO series	Platform	Gene chip	Normal (*n*)	ESCA (*n*)	Experiment type
GSE1420	GPL96	HG-U133 A	8	8	Array
GSE20347	GPL571	HG-U133 A	17	17	Array

**Table 2:** Clinical characteristics of ESCA patients.

Characteristic	*n* (%)
Age	
<55 years	52 (28.42)
≥55 years	131 (71.58)

Gender	
Female	27 (14.75)
Male	156 (85.25)

Survival status	
Death	76 (41.53)
Survival	107 (58.47)

Alcohol consumption history	
Yes	128 (69.95)
No	55 (30.05)

Radiation therapy	
Yes	48 (26.23)
No	135 (73.77)

Pharmaceutical	
Yes	42 (22.95)
No	141 (77.05)

*T* classification	
*T*0	2 (1.09)
*T*1	32 (17.49)
*T*2	44 (24.04)
*T*3	100 (54.64)
*T*4	5 (2.73)

*N* classification	
*N*0	79 (43.17)
*N*1	73 (39.89)
*N*2	21 (44.48)
*N*3	8 (4.37)
*NX*	2 (1.09)

Stage	
I	14 (7.65)
II	92 (50.27)
III	65 (35.52)
IV	12 (6.56)

Histological grade	
*G*1	19 (10.38)
*G*2	76 (41.53)
*G*3	48 (26.23)
*GX*	40 (21.86)

*M* classification	
*M*0	148 (80.87)
*M*1	12 (6.56)
*M*X	23 (12.57)

**Table 3:** Univariate analysis and multivariate analysis of the correlation of EpCAM expression with OS among ESCA patients.

Parameter	Univariate analysis			Multivariate analysis		
	HR	95% CI	*P*	HR	95% CI	*P*
Age	1.00	0.98–1.03	0.731	1.02	0.98–1.05	0.323
Gender	4.23	1.02–17.47	**0.046**	2.15	0.49–9.46	0.310
Histologic grade	1.48	0.92–2.36	0.102	0.94	0.53–1.66	0.821
Stage	2.51	1.68–3.75	**7.40E 06**	1.73	0.57–5.27	0.336
*T* classification	1.32	0.90–1.94	0.159	1.14	0.66–1.96	0.633
*M* classification	3.64	1.60–8.28	**0.002**	1.87	0.27–13.09	0.529
*N* classification	1.74	1.27–2.39	**0.001**	1.34	0.72–2.46	0.363
EpCAM	1.00	1.00–1.01	**0.004**	1.00	1.00–1.01	**0.010**

Bold values indicate *P* < 0.05. HR, hazard ratio; CI, confidence interval.

**Table 4:** The 2D protein-ligand interaction.

No.	Compound name	CAS	Score	Hydrogen bond	Hydrophobic interactions
1	Ginsenoside Rg3	14197-60-5	5.91	Lys151, Lys221	Asp232, Leu242, Val220, Pro244, Gly222, Asp219, Phe216, Ile193, Glu169, Ile170, Leu166, Tyr174, Leu233
2	Ginsenoside Rh2	78214-33-2	5.24	Leu240	Glu169, Ile170, Leu233, Asp232, Asp241, Leu242, Pro244, Val220, Lys229, Asp219, Arg173, Phe216, Tye174, Leu166
3	Ginsenoside Rg5	186763-78-0	4.10	Lys151, Asp241	Tyr174, Glu169, Leu233, Leu242, Asp219, Asp232, Arg173, Pro244, Val220, Leu240, Ile170, Phe216, Leu148
4	Ginsenoside Rk3	364779-15-7	3.59	Lys151, Asp241	Gly222, Lys229, Arg173, Asp219, Asp232, Leu240, Leu233, Val220, Leu242, Pro244

## Data Availability

The data used to support the findings of this study are included within the supplementary information file.

## Supplementary Materials

Supplementary Materials Figure S1: MMP1, SPP1, and CRNN expression in ESCA. Figure S2: multivariate analysis of the correlation of EpCAM expression with OS among ESCA patients. Figure S3: structure of interaction between ginsenoside and EpCAM.
