# Supplementary material for: Study on the Selection of the Targets of Esophageal Carcinoma and Interventions of Ginsenosides Based on Network Pharmacology and Bioinformatics
Source: Evid Based Complement Alternat Med. 2020 Jun 24;2020:4821056. doi: 10.1155/2020/4821056 (PMC7333027; doi:10.1155/2020/4821056)
Supplement: Supplementary Materials — Figure S1: MMP1, SPP1, and CRNN expression in ESCA. Figure S2: multivariate analysis of the correlation of EpCAM expression with OS among ESCA patients. Figure S3: structure of interaction between ginsenoside and EpCAM. [file 4821056.f1.pdf]

Study on the selection of the targets of esophageal carcinoma and interventions of Ginsenosides based on network pharmacology and bioinformatics

Xin Yang, Yahui Li, Haibing Qian*

Guizhou University of Traditional Chinese Medicine, Guiyang, 550025, PR China

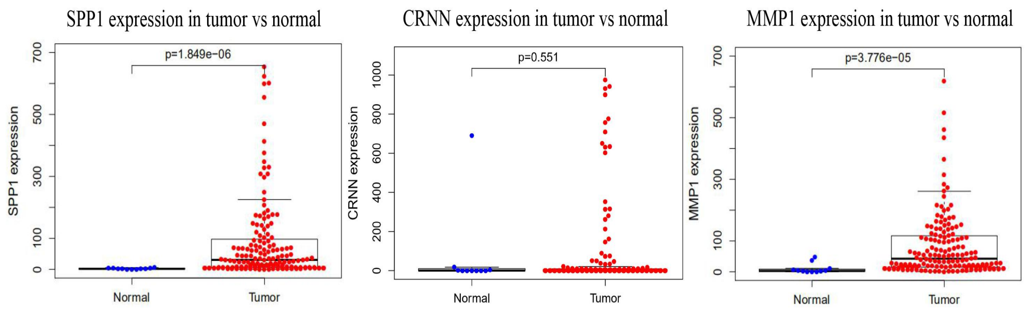

Figure S1: MMP1, SPP1 and CRNN expression in esophageal carcinoma (Blue-low expression; Red-high expression).

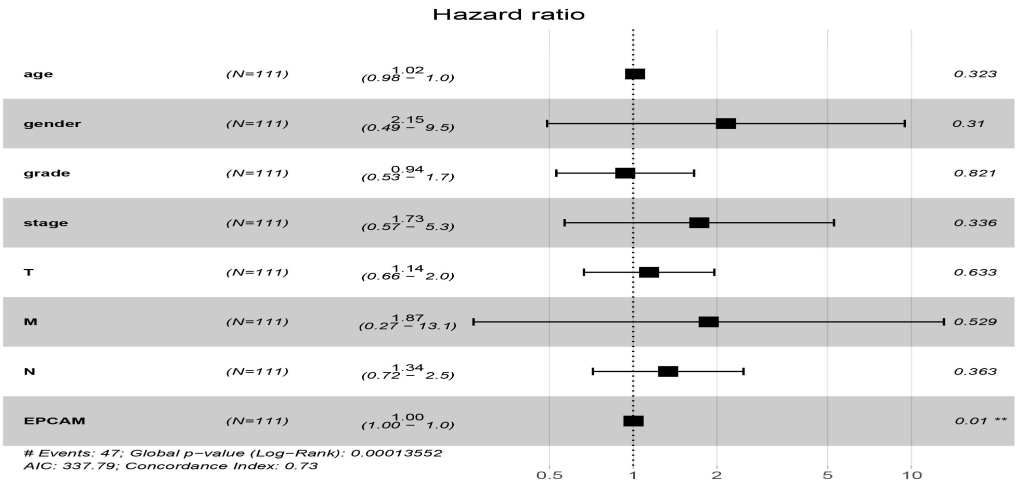

Figure S2: Multivariate analysis of the correlation of EpCAM expression with OS among esophageal carcinoma patients

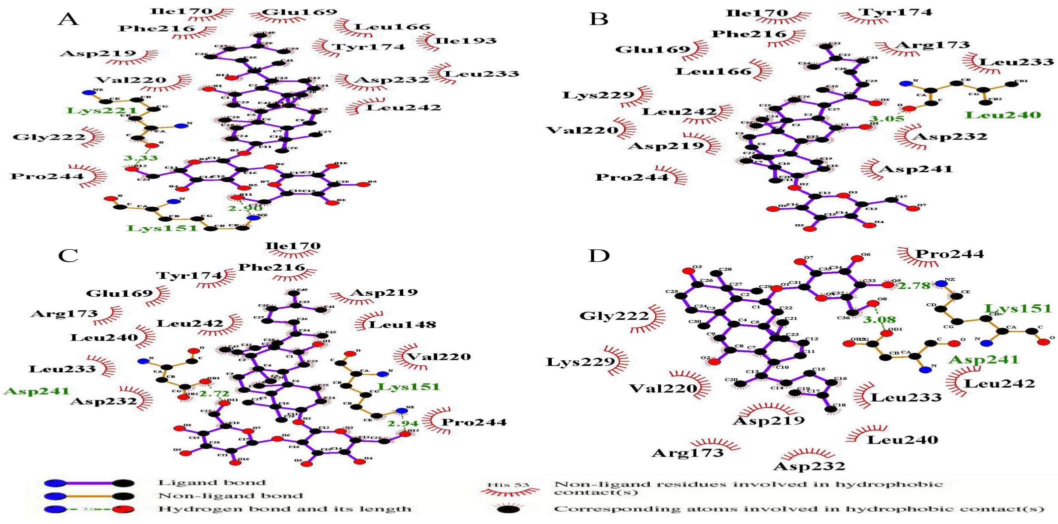

Figure S3:Structure of interaction between Ginsenosides and EpCAM. (A) Ginsenoside Rg3, (B) Ginsenoside Rh2, (C) Ginsenoside Rg5, (D) Ginsenoside Rk3.
